# Supplementary material for: Role and mechanism of NCAPD3 in promoting malignant behaviors in gastric cancer
Source: Front Pharmacol. 2024 Apr 22;15:1341039. doi: 10.3389/fphar.2024.1341039 (PMC11070777; doi:10.3389/fphar.2024.1341039)
Supplement: Supplementary file 11 [file DataSheet2.ZIP › GSEA/immunologic/my_analysis.Gsea.1599462003817/GSE39820_CTRL_VS_IL1B_IL6_IL23A_CD4_TCELL_DN.html]

Details for gene set GSE39820\_CTRL\_VS\_IL1B\_IL6\_IL23A\_CD4\_TCELL\_DN[GSEA]

|  || Dataset | filtered\_dataset.sample\_info.cls#WT\_versus\_NCAPD3\_MUT |
| Phenotype | sample\_info.cls#WT\_versus\_NCAPD3\_MUT |
| Upregulated in class | NCAPD3\_MUT |
| GeneSet | GSE39820\_CTRL\_VS\_IL1B\_IL6\_IL23A\_CD4\_TCELL\_DN |
| Enrichment Score (ES) | -0.4939972 |
| Normalized Enrichment Score (NES) | -2.4473262 |
| Nominal p-value | 0.0 |
| FDR q-value | 0.014899823 |
| FWER p-Value | 0.167 |
Table: GSEA Results Summary

  

Fig 1: Enrichment plot: GSE39820\_CTRL\_VS\_IL1B\_IL6\_IL23A\_CD4\_TCELL\_DN      
 Profile of the Running ES Score & Positions of GeneSet Members on the Rank Ordered List

  

| SYMBOL | TITLE | RANK IN GENE LIST | RANK METRIC SCORE | RUNNING ES | CORE ENRICHMENT || 1 | 2120 | ETV6 | 63 | 0.862 | 0.0138 | No |
| 2 | 4193 | MDM2 | 131 | 0.769 | 0.0183 | No |
| 3 | 90025 | UBE3D | 167 | 0.710 | 0.0418 | No |
| 4 | 85315 | PAQR8 | 351 | 0.567 | -0.0512 | No |
| 5 | 839 | CASP6 | 792 | 0.330 | -0.3457 | No |
| 6 | 4502 | MT2A | 863 | -0.277 | -0.3772 | No |
| 7 | 54498 | SMOX | 965 | -0.384 | -0.4236 | No |
| 8 | 51646 | YPEL5 | 1014 | -0.413 | -0.4298 | No |
| 9 | 3340 | NDST1 | 1104 | -0.481 | -0.4609 | Yes |
| 10 | 7378 | UPP1 | 1111 | -0.484 | -0.4320 | Yes |
| 11 | 49854 | ZBTB21 | 1118 | -0.493 | -0.4025 | Yes |
| 12 | 55124 | PIWIL2 | 1154 | -0.516 | -0.3923 | Yes |
| 13 | 1032 | CDKN2D | 1156 | -0.519 | -0.3573 | Yes |
| 14 | 831 | CAST | 1160 | -0.524 | -0.3235 | Yes |
| 15 | 665 | BNIP3L | 1185 | -0.556 | -0.3027 | Yes |
| 16 | 5154 | PDGFA | 1191 | -0.566 | -0.2674 | Yes |
| 17 | 4651 | MYO10 | 1213 | -0.588 | -0.2421 | Yes |
| 18 | 26999 | CYFIP2 | 1234 | -0.611 | -0.2146 | Yes |
| 19 | 1893 | ECM1 | 1250 | -0.637 | -0.1816 | Yes |
| 20 | 2355 | FOSL2 | 1262 | -0.648 | -0.1450 | Yes |
| 21 | 1649 | DDIT3 | 1341 | -0.779 | -0.1477 | Yes |
| 22 | 10397 | NDRG1 | 1380 | -0.925 | -0.1116 | Yes |
| 23 | 64764 | CREB3L2 | 1382 | -0.930 | -0.0484 | Yes |
| 24 | 2040 | STOM | 1389 | -0.988 | 0.0151 | Yes |
Table: GSEA details [plain text format]

  

Fig 2: GSE39820\_CTRL\_VS\_IL1B\_IL6\_IL23A\_CD4\_TCELL\_DN      
 Blue-Pink O' Gram in the Space of the Analyzed GeneSet

  

Fig 3: GSE39820\_CTRL\_VS\_IL1B\_IL6\_IL23A\_CD4\_TCELL\_DN: Random ES distribution      
 Gene set null distribution of ES for **GSE39820\_CTRL\_VS\_IL1B\_IL6\_IL23A\_CD4\_TCELL\_DN**
